# Cold tolerance triggered by soluble sugars: a multifaceted countermeasure

**DOI:** 10.3389/fpls.2015.00203

**Published:** 2015-04-14

**Authors:** Łukasz P. Tarkowski, Wim Van den Ende

**Affiliations:** Laboratory of Molecular Plant Biology, Katholieke Universiteit LeuvenLeuven, Belgium

**Keywords:** cold tolerance, RFO, fructans, sugar signaling, DELLA

In their natural habitat, plants are continuously challenged by adverse environmental conditions. Among them, cold stress (CS) is a major environmental factor limiting agricultural productivity and geographic distribution (Chinnusamy et al., 2007). In this respect, chilling (15-0°C) and freezing (<0°C) stress should be distinguished (Thomashow, 2010). CS responses at the cellular level are characterized by an extensive reprogramming of gene expression and metabolic fluxes (Stitt and Hurry, 2002; Miura and Furumoto, 2013). Clearly, these modifications are mainly linked to the onset of tolerance mechanisms, which ultimately lead to acclimation. Several metabolites are known to contribute to this process, including amino acids, polyamines, polyols, and soluble sugars (Krasensky and Jonak, 2012 and references therein). Among them, particular focus was recently given to understand the multifunctional role of soluble sugars in enhancing cold tolerance (Nägele and Heyer, 2013).

Accumulation of soluble sugars following CS is known since long (Levitt, 1958), including studies on their potential roles in stabilizing biological components, particularly for Raffinose Family Oligosaccharides (RFO) (Santarius, 1973). Despite this well-known correlation, more recent investigations shed light on the potential underlying biological mechanisms involved (Valluru et al., 2008; Sicher, 2011; Peng et al., 2014). One of the major factors affecting overall cellular stability under CS is membrane phospholipid composition regulating membrane fluidity (Ruelland and Collin, 2011 and references therein) associated with cold stimulus perception, as suggested by the protein kinases cascade activation triggered by dimethyl sulfoxide (DMSO)-mediated membrane rigidification (Furuya et al., 2014). Different saccharides are capable to directly stabilize biological membranes under stress conditions. Sucrose (Suc) can directly protect cell membranes by interacting with the phosphate in their lipid headgroups, decreasing membrane permeability (Strauss and Hauser, 1986). Fructans, fructose-based oligo- and polysaccharides, and RFO can increase stability of phospholipidic mono- and bilayers by direct insertion between polar headgroups (Vereyken et al., 2001; Hincha et al., 2003). Fructans are localized in the vacuole, suggesting that their contribution to membrane stabilization may be restricted to the tonoplast. However, their detection in the apoplast of cold-stressed plants also suggests a role in the protection of the plasma membrane, where they can be delivered by a vesicle-mediated transport (Valluru et al., 2008). This scenario seems to be different for RFO. Despite their cytosolic biosynthesis, their protective action may be restricted to chloroplast inner membranes, as suggested by research on *Arabidopsis thaliana* (Nägele and Heyer, 2013 and references therein). Thus, specific changes in subcellular concentrations of potential stress protectants may greatly influence successful responses (Lunn, 2007) (Figure 1). The regulation of the activity and/or expression of soluble sugar transporters, especially those involved in chloroplast and Tonoplast Monosaccharide Transporters (TMTs) (Wormit et al., 2006) and Sugars Will Eventually Be Exported Transporters (SWEETs) (Klemens et al., 2013), may play a central role in such processes. Cold-stressed *AtSWEET16* overexpression lines showed increased freezing tolerance and increased glucose (Glc) and Suc levels (Klemens et al., 2013). The fructose (Fru)-specific transporter AtSWEET17 plays a primary role in Fru homeostasis following 1-week 4°C treatment (Guo et al., 2014b). These authors suggested that the Fru-specific transport features of this carrier may be mediated by a Fru-specific signaling pathway. Taken together, these works indicate that the activity and/or expression of sugars transporters may be regulated by sugar signaling, affecting the subcellular distribution of sugars and overall cellular sugar homeostasis, which may be tightly linked to the cellular redox homeostasis (see next paragraph). In that respect, it will be particularly interesting to characterize the nature of the Raffinose importer in the chloroplast (Schneider and Keller, 2009) and to decipher its activation by sugar- and hormone signaling under CS in tolerant accessions.

**Figure 1:**
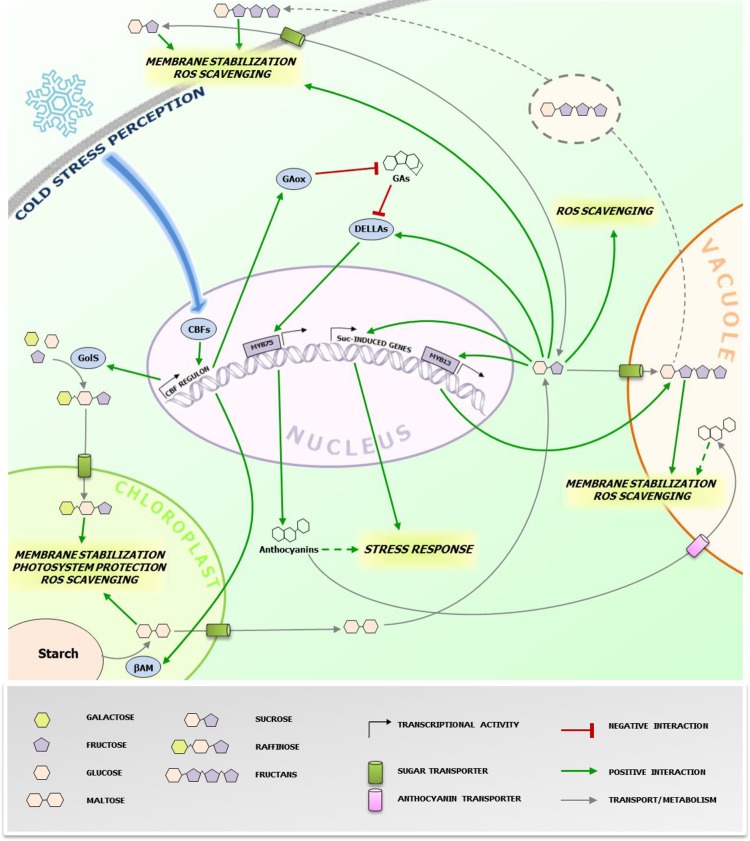
**Protective effects of cold-induced saccharides at the subcellular level**. The figure highlights the (putative) action sites of sugars accumulating during CS responses in higher plants cells. The grey dotted line refers to the proposed vesicular transport mechanism of fructans from the vacuole to the plasma membrane in fructan accumulating species (Valluru et al., 2008). The green dotted line refers to the possible roles of anthocyanins in CS protection. Anthocyanins are also imported in the vacuole through ABC class transporters (Francisco et al., 2013), where they can contribute in alleviating CS. The blue arrow represents the signaling pathway leading to the activation of CBFs. The biosynthesis and metabolic conversions of the sugars involved is oversimplified and represented by grey arrows. CBFs, C-repeat binding factors; GAs, gibberellins; GAox, GA oxidase; GolS, galactinol synthase; βAM, β-amylase; Suc, sucrose. Specific effects of different sugars/anthocyanins are highlighted in italic. Readers are referred to the figure legend and the text for further details.

Reactive oxygen species (ROS) production partially contributes to chilling and freezing damage (Nishizawa et al., 2008 and references therein). Recently, several carbohydrates were proposed as important components of the cellular ROS scavenging system, perhaps in synergism with other components such as phenylpropanoids (Couée et al., 2006; Nishizawa et al., 2008; Van den Ende and Valluru, 2009). Living cells do not possess an efficient enzymatic system to scavenge the highly deleterious hydroxyl radical (·OH) (Gechev et al., 2006). Furthermore, carbohydrates have generally higher scavenging ability against ·OH as compared with other radicals, such as superoxide (O^·−^_2_) (Stoyanova et al., 2011). The recent works of Peshev et al. (2013) and Peukert et al. (2014) provided new mechanistic insights into this process. They observed that Fenton reaction-derived ·OH scavenging by fructans *in vitro* lead to the formation of new oligosaccharides and oxidized sugars. Such oxidized sugars can also be found *in vivo*, suggesting that fructans function as scavengers *in planta* (Peukert et al., 2014). Alternatively or additionally, fructans have been proposed as stress signals, further amplifying stress responses that may be initiated by Suc-specific signaling pathways (Van den Ende, 2013).

Contrary to fructans, the signaling capacity of small metabolic sugars is widely recognized (Ramon et al., 2008; Ruan, 2014) and several lines of evidence indicates their involvement in regulating various stress responses (Van den Ende and El-Esawe, 2014). Recently, a possible mechanistic link between sugars and CS tolerance was proposed by Peng et al. (2014). They successfully expressed *PtrBAM1*, a stress-responsive chloroplastic β-amylase-coding gene from *Poncirus trifoliata* in tobacco, under the constitutive promoter CaMV35S. They found that β-amylase activity was strongly enhanced by CS accompanied with a massive accumulation of maltose and other soluble sugars (Figure 1). Importantly, this breakthrough paper provides the first evidence that PtrCBF1 (C-repeat-binding factor 1), a transcription factor belonging to a family of central regulators of CS responses highly conserved throughout plants kingdom (Chinnusamy et al., 2007), can bind directly to the promoter of *PtrBAM1*, providing a unique link between CBF-mediated cold responses and sugar dynamics. Thus, cold-dependent sugar accumulation may, at least partially, depend on the CBF transcriptional cascade, as previously suggested by the CBF-dependent metabolic changes observed during CS (Cook et al., 2004).

It is noteworthy that Suc can trigger fructan synthesis and accumulation in fructan accumulators such as wheat, by activating the transcriptional factor TaMYB13, which directly controls gene expression of enzymes involved in fructan synthesis (Kooiker et al., 2013). A possible scenario for future research could be that CBF-dependent increases of Suc under CS may trigger fructan synthesis and accumulation in wheat and other fructan accumulators, allowing a highly coordinated metabolic countermeasure onset, via an orchestration of direct and indirect signaling and scavenging mechanisms, as proposed above (Figure 1). Accordingly, in winter wheat, fructans accumulate in young plants during cold acclimation in the autumn, and this process is also associated with increased snow-mold resistance (Yoshida et al., 1998). The high correlation between fructan accumulation and cold tolerance in the wheat family was recently confirmed by studies on artificially obtained wheat hexaploid lines characterized by different degrees of freezing tolerance (Yokota et al., 2015). In line with these views, it has been shown that transgenic rice plants carrying wheat fructosyltransferase (FT) genes showed an increased CS tolerance (Kawakami et al., 2008).

Galactinol synthase (GolS), the enzyme catalyzing the first step in RFO biosynthesis, is considered as a target gene of the CBF regulon (Taji et al., 2002), leading to RFO accumulation under CS. Notably, galactinol, and raffinose have also been proposed as important signals during biotic interactions (Kim et al., 2008). Another emerging point of convergence between CBFs and sugars is that AtCBF1 enhances accumulation of DELLA proteins, fundamental repressors of gibberellin (GA) signaling and positive regulators of stress responses (Claeys et al., 2014), by stimulating GA catabolism through increased expression of GA2-oxidase genes (Achard et al., 2008). Furthermore, it has been recently demonstrated that DELLAs can be specifically stabilized by Suc, but not by Glc (Li et al., 2014). DELLA proteins stimulate anthocyanin synthesis through activation of the PAP1/MYB75 transcription factor (Li et al., 2014). In general anthocyanin levels positively correlate with cold tolerance (Janska et al., 2010), probably by protecting chlorophyll from over-excitement under freezing conditions (Hannah et al., 2006).

Soluble sugars levels are strictly connected with starch synthesis and breakdown dynamics, which are on their turn tightly regulated by the circadian clock (Graf et al., 2010). In turn, sugar levels have fundamental roles in entraining the clock (Haydon et al., 2013). Cold-responsive genes such as *AtCBF1* show diurnal oscillations in their expression (Nakamichi et al., 2009). Moreover, expression of central clock components and diurnal regulated genes is largely influenced by CS (Miura and Furumoto, 2013), providing tight connections between the clock, metabolic adjustments and CS responses. Recently, Sicher (2011) shed light on the importance of starch dynamics during chilling responses in Arabidopsis, by comparing starch and different sugar profiles during chilling stress in light/dark conditions in wild-type and *pgm1* starchless mutants. This author demonstrates that synthesis and accumulation of the two most highly induced sugars during chilling stress, maltose and raffinose, strictly depend on the presence of starch, demonstrating the intimate interconnection between RFO, sucrose, and starch metabolisms (Figure 2). It is known that both target of rapamycin (TOR) and SnRK1 kinases influence such processes (Dobrenel et al., 2013), but the exact underlying mechanisms need further exploration. Future research on crop species under CS should focus on the dynamics of *all* carbohydrate pools in a diurnal context, to be able to better understand the complete picture.

**Figure 2:**
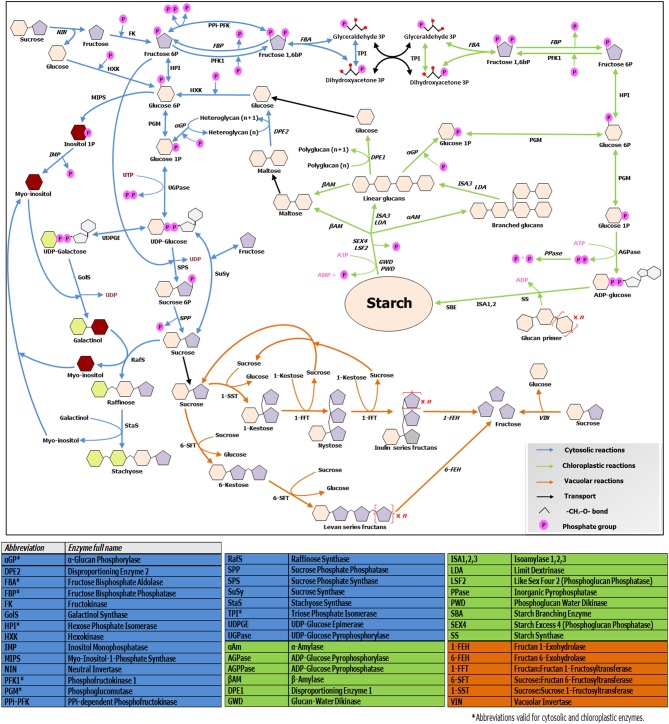
**Overview of soluble sugars, RFO, fructans and starch metabolic pathways**. The scheme illustrates connections and divergences between the above mentioned pathways, taking in account their different subcellular environments. Enzymes involved in catabolic steps are written in italic. Note that only the metabolism of linear fructans is illustrated, due to space constraints. For further details, readers are referred to http://plantsinaction.science.uq.edu.au/book/export/html/121. for starch and sucrose metabolism; and to Vijn and Smeekens (1999) and Nishizawa et al. (2008) for more details on fructan and raffinose biosynthesis, respectively.

Besides the CRB signaling pathway, which is necessary but not sufficient to trigger cold acclimation, chilling, and freezing tolerance, several phytohormones also play critical roles by positively or negatively influencing cold resistance and acclimation (Thomashow, 2010; Miura and Furumoto, 2013). Among them, ethylene and abscisic acid (ABA) stand out for their well-known crosstalk with sugar signaling pathways (Gazzarrini and McCourt, 2001). Sugar signaling was also shown to take part in stress responses, and this is particularly evident when Suc-specific responses are involved (Van den Ende and El-Esawe, 2014), including the upregulation of the phenylpropanoid biosynthetic pathway (Serrano et al., 2012; Li et al., 2014), with a strong impact on the anthocyanin biosynthetic branch (Teng et al., 2005). As in development, during CS response the ABA-ethylene dynamics conserve an antagonistic nature, with CBF1 as major crosstalk point (Thomashow, 2010; Shi et al., 2012). Both ABA and Suc promote the accumulation of DELLA proteins (Guo et al., 2014a; Li et al., 2014), urging further research on possible ABA-sugar signaling synergisms under CS. Intriguingly, the accumulation of DELLA proteins is also mediated by CBF1 through posttranslational mechanisms, which seems to be required for the full activation of freezing tolerance in *A. thaliana* (Achard et al., 2008). Thus, it can be speculated that DELLA proteins play an important role in orchestrating ABA and sugar-induced CS responses, but this requires further research. This idea was proposed even in a much broader context by De Bruyne et al. (2014), considering DELLA proteins as pivotal modulators of the physiological balance between growth and overall (also biotic) stress responses by integrating sugar and hormonal inputs.

Thanks to their biochemical properties and availability, sugars are likely to be used by plants in counteracting the most commonly occurring adversities in their natural environment. Overall, modulation of compatible solutes, among which sugars typically represent a vast majority, may represent one of the basic mechanisms involved in multistress tolerance (Puniran-Hartley et al., 2014). Plant responses to evolutionary pressures in stressful environments led to the diversification of sugar structures and functions, as well represented by the RFO and fructan cases, among other oligosaccharides (Van den Ende, 2013). Furthermore, the ability of sugars to modulate expression of stress-related genes involved in both abiotic and biotic stress responses, such as phenylalanine ammonia lyase and pathogenesis related proteins (Herbers et al., 1996; Barau et al., 2014), testifies the high integration level of carbohydrates in cellular defensive strategies. Moreover, it has been demonstrated that sugar dynamics in the apoplastic environment need to be dissected from those occurring within the cells, a very important notion for future research (Barau et al., 2014). Recent data strongly support the involvement of invertases, key controllers of compartment-specific Suc/hexose ratios, in response to both biotic and abiotic stresses (Albacete et al., 2014; Sun et al., 2014). An important goal for future research will be to unravel how invertases and other Suc metabolizing enzymes are precisely connected to the main stress signaling pathways, and in which way they influence growth/defense balances, intimately connected to TOR and SnRK1 activities. In the coming years, dissection of stress-specific signaling pathways initiated by sugar signaling will likely become one of the most exciting topics in plant physiology, disclosing new possibilities to increase multistress tolerance in crops.

## Conflict of interest statement

The authors declare that the research was conducted in the absence of any commercial or financial relationships that could be construed as a potential conflict of interest.
